# Rare Metastasis to the Submandibular Gland in Oral Squamous Cell Carcinoma

**DOI:** 10.3389/fonc.2021.728230

**Published:** 2021-11-26

**Authors:** Ping Zhou, Jing-Xin Chen, Yuan Zhou, Chen-Lu Lian, Bing Yan, San-Gang Wu

**Affiliations:** ^1^ Department of Radiation Oncology, The First Affiliated Hospital of Xiamen University, Xiamen, China; ^2^ Department of Stomatology, Hainan General Hospital (Hainan Affiliated Hospital of Hainan Medical University), Haikou, China; ^3^ Department of Otolaryngology Head and Neck Surgery, The First Affiliated Hospital of Xiamen University, Xiamen, China

**Keywords:** oral squamous cell carcinoma, submandibular gland, organ preservation, level IB metastasis, head and neck cancer

## Abstract

**Purpose:**

In the current recommendation of neck dissection in oral squamous cell carcinoma (OSCC), the submandibular gland (SMG) should also be removed. This study aimed to investigate the incidence and the patterns of SMG involvement in OSCC patients.

**Methods:**

Patients initially diagnosed with OSCC between January 2018 and October 2020 were included. The distribution of lymph nodes metastasis in level IB was analyzed.

**Results:**

We included 145 patients who underwent primary surgery and neck dissection in this study. All patients had level IB lymph node dissection and simultaneous removal of the SMG. Of these patients, only one patient (0.7%) had involvement in SMG by directly infiltrating from the primary tumor. A total of 18 positive lymph nodes were found in level IB in 16 patients, and no positive lymph nodes were located in the SMG. There were 6 lymph nodes located in the lateral part of the SMG and 12 lymph nodes located in the anterior of the SMG. Patients with tumors located in the buccal mucosa and N3 stage were the independent predictive factors associated with level IB nodal metastasis.

**Conclusion:**

Involvement of SMG in OSCC is quite rare. Preservation of the SMG during neck dissection in selected patients with OSCC seems to be feasible and oncologically safe.

## Introduction

According to the GLOBOCAN 2020, cancers developed in the lip and oral cavity accounted for approximately 2% of all cancers in the world, with over 370,000 cases newly diagnosed with lip and oral cavity cancers and 170,000 disease-related deaths occurring annually (1). The majority of oral cavity cancers are squamous cell carcinoma (SCC) (2). Approximately 29%–36% of oral squamous cell carcinoma (OSCC) patients had cervical lymph node involvement (3, 4). In patients with early-stage (T1) and clinically lymph node-negative disease, 23% of them had occult lymph node metastasis during neck dissection (5). Therefore, primary surgery and neck dissection remain the most important management for OSCC (6).

The submandibular gland (SMG), which is located in the submandibular triangle, has the predominant function of saliva secretion. According to the 2013 edition of the neck nodal classification in the neck, SMG is one of the contents of level IB (7). A large case series from a literature review included 2,750 patients with OSCC, and only 2 patients (0.07%) had intraglandular lymph node metastases (8). In addition, the probability of direct involvement to SMG by primary tumor or periglandular nodal extension through the capsule was only 0%–4.5% (8). Moreover, the prior study also showed comparable survival outcomes between the SMG preservation group and the removal group (9). However, in the current clinical practice, SMG excision is a regular part of level IB dissection in OSCC. In this study, we aimed to investigate the incidence and risk factors of SMG involvement in OSCC patients, which could add to the knowledge regarding the preservation of SMG in this patient subset.

## Materials and Methods

### Data Collection and Patient’s Selection Criteria

We retrospectively included patients diagnosed with OSCC between January 2018 and October 2020. Patients who met the following criteria were included in this study: (1) histopathologically confirmed SCC, (2) primary tumor located in the oral cavity, (3) received primary tumor resection and ipsilateral with or without contralateral neck dissection, and (4) removal of ipsilateral level IB and simultaneous removal of the SMG. All cases of OSCC were confirmed by histopathology. Patients who received preoperative chemotherapy, preoperative radiotherapy, or preoperative chemoradiotherapy were excluded. The study was approved by the Institutional Review Board of the First Affiliated Hospital of Xiamen University (approval number: XMYY-2021KY052). Written informed consent for participation was not required for this study in accordance with the national legislation and the institutional requirements.

### Measures

OSCC in our institution was generally treated with primary surgical resection with concomitant neck dissection. All patients received standard neck dissection due to the higher incidence of occult nodal metastasis in OSCC (10, 11). The extent of neck dissection included a minimum of levels I–III with SMG resection in all cases. Bilateral neck dissection was conducted in those with tumors involving or approaching the midline. The following clinicopathologic characteristics were identified, including gender, age, primary tumor sites, smoking use, alcohol use, tumor grade, tumor (T) stage, nodal (N) stage, American Joint Committee on Cancer (AJCC) stage, surgery margin status, and the details of neck dissection. Slides stained with hematoxylin and eosin were assessed to confirm the diagnosis and to perform histopathological grading of the tumors based on the adaptation from Bryne et al. (12). The distribution of lymph node involvement in level IB and around the SMG was analyzed. The eighth edition of the AJCC staging was used in this study, which integrated the depth of invasion and extranodal extension into the tumor–node–metastasis (TNM) classification systems, respectively (13).

### Statistical Analysis

The logistic regression analysis was performed to identify predictive factors associated with level IB lymph node metastasis. SPSS statistical software (version 25.0, IBM Corporation, Armonk, NY, USA) was used for data analysis. *p* < 0.05 was considered to be statistically significant.

## Results

### Patients’ Clinicopathological Characteristics

A total of 145 patients were identified in this study, namely, 96 males (66.2%) and 49 females (33.8%). The median age was 60 years (range, 27–83 years). **Table 1** lists the baseline characteristics of patients. Of these patients, 106 (73.1%), 20 (13.8%), 9 (6.2%), and 7 (4.8%) had tumors developed in the tongue, buccal mucosa, the floor of the mouth, and gingiva, respectively. In patients with available tumor grade (*n* = 143), moderately differentiated disease predominated with 76.2% (*n* = 109), and 11.9% (*n* = 17) and 11.9% (*n* = 17) of them were poorly differentiated and well-differentiated, respectively. There were 49 (33.8%), 64 (44.1%), 54 (14.5%), and 11 (7.6%) patients who had stage T1, T2, T3, and T4 diseases, respectively. A total of 117 (80.7%) patients underwent ipsilateral neck dissection and 28 (19.3%) underwent bilateral neck dissection. Sixty-one patients (42.1%) were pathologically diagnosed with lymph node metastases, including 11 (18.0%), 36 (59.0%), and 14 (23.0%) patients who had stage N1, N2, and N3 diseases, respectively. According to the 8th TNM staging, there were 38 (26.2%), 34 (23.4%), 20 (13.8%), and 53 (36.6%) patients who were pathologically diagnosed with stage I, II, III, and IVA diseases, respectively. Most of the patients (95.9%) had negative surgical margins.

**Table 1:** Patient characteristics.

Variables	Number (%)
Gender	
Male	96 (66.2)
Female	49 (33.8)
Age	
<50 years	33 (22.8)
≥50 years	112 (77.2)
Primary site	
Lip	1 (0.7)
Upper jaw	1 (0.7)
Buccal mucosa	20 (13.8)
Mouth floor	9 (6.2)
Retromolar trigone	1 (0.7)
Tongue	106 (73.1)
Gingiva	7 (4.8)
Smoking pack-year index	
0	81 (55.9)
<20	17 (11.7)
≥20	47 (32.4)
Alcohol use	
Never	69 (47.6)
Normal	45 (31.0)
Abuse	31 (21.4)
Tumor grade	
Well differentiation	17 (11.7)
Moderate differentiation	109 (75.2)
Poor differentiation	17 (11.7)
Unknown	2 (1.4)
Tumor stage	
T1	49 (33.8)
T2	64 (44.1)
T3	21 (14.5)
T4	11 (7.6)
Nodal stage	
N0	84 (57.9)
N1	11 (7.6)
N2	36 (24.8)
N3	14 (9.7)
AJCC stage	
I	38 (26.2)
II	34 (23.4)
III	20 (13.8)
IVA	53 (36.6)
Margin status	
Negative	139 (95.9)
Positive	6 (4.1)
Neck dissection	
Ipsilateral	117 (80.7)
Bilateral	28 (19.3)
Submandibular gland involved	
No	144 (99.3)
Yes	1 (0.7)

AJCC, American Joint Committee on Cancer; T, tumor; N, nodal.

Among all patients, 76 (52.4%) had a history of alcohol use, including 31 (40.8%, 31/76) patients with a history of alcohol abuse. In patients with alcohol abuse, the median daily Chinese Baijiu consumption was 150 ml (range, 50–700 ml), and the median alcohol intake time was 30 years (range, 2–40 years). In addition, there were 64 (44.1%) patients who had a history of smoking, the median smoking intensity was 20 (range, 2–60) cigarettes per day, the median smoking time was 30 years (range, 5–50 years), and the median smoking index was 30 pack-years (range, 2–120 pack-years).

### SMG Invasion

**Figure 1:**
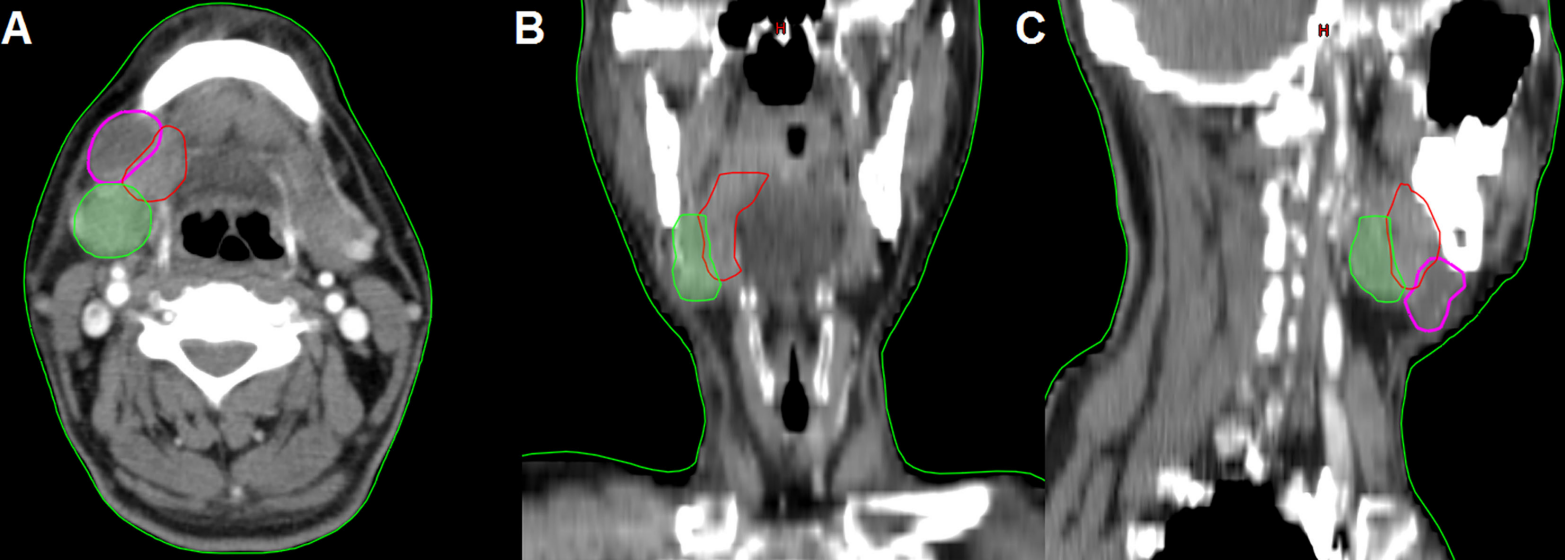
The preoperative computed tomography image of submandibular gland involvement in axial **(A)**, coronal **(B)**, and sagittal views **(C)** (red, primary tumor; purple, lymphadenopathy in level IB; green, submandibular gland).

Only one patient (0.7%) with stage IVA disease and primary tumor located in the tongue had involvement of the SMG. The SMG was involved by direct infiltration from the ventral tongue (**Figure 1**). This patient was a 57-year-old man who was clinically diagnosed with T4N2bM0 oral tongue cancer. The maximum diameter of the primary tumor was 6.8 cm. Ipsilateral neck dissection was performed. There were 36 lymph nodes that were dissected and 2 were metastasized. A preoperative computed tomography scan showed that the SMG was directly infiltrated by the primary tumor.

### Peri-Submandibular Node Involvement

**Figure 2:**
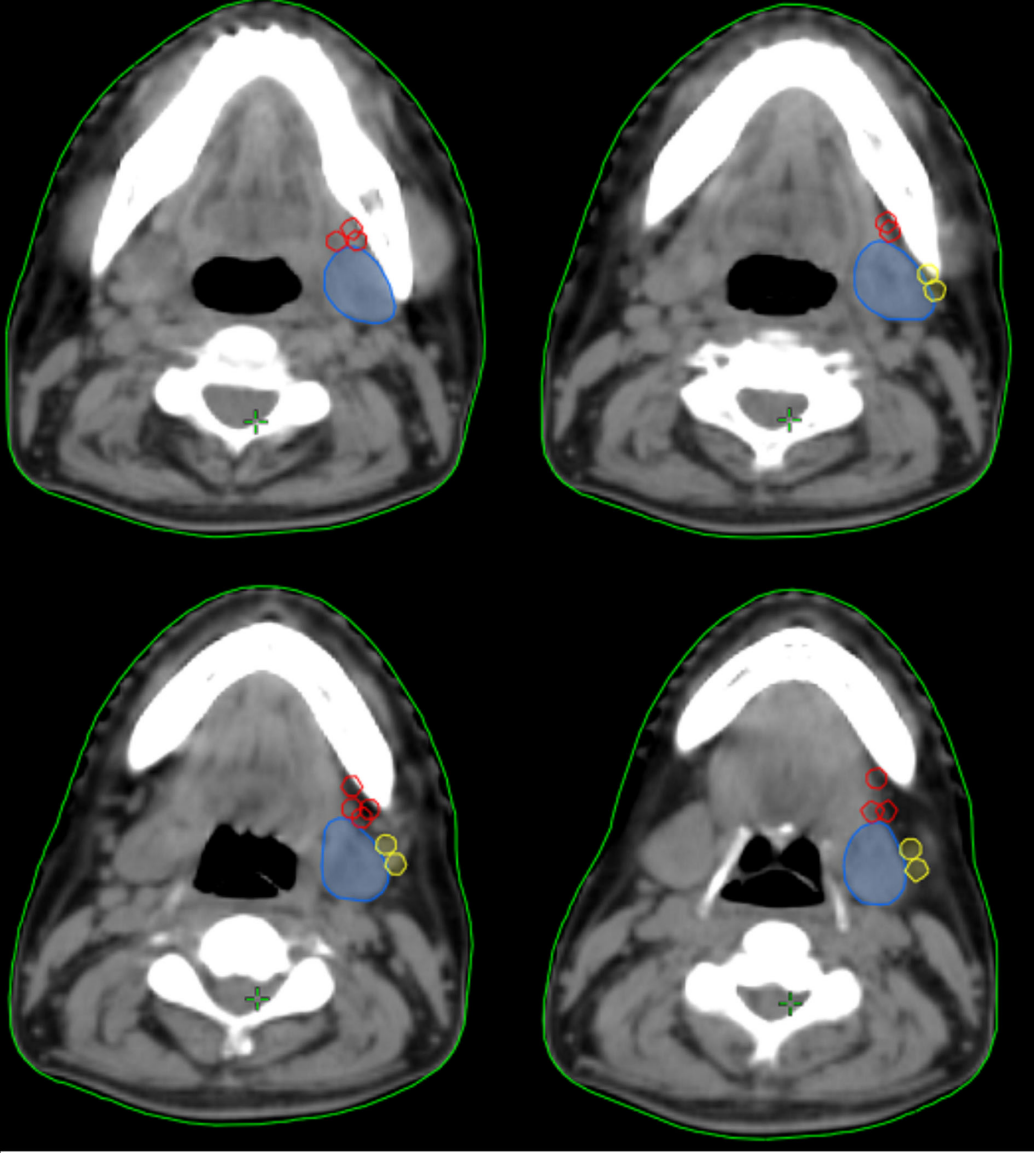
The patterns of peri-submandibular lymph node metastases in oral squamous cell carcinoma (blue, submandibular gland; red, lymph node metastases in the anterior part of the submandibular gland; yellow, lymph node metastases in the lateral part of the submandibular gland).

**Figure 3:**
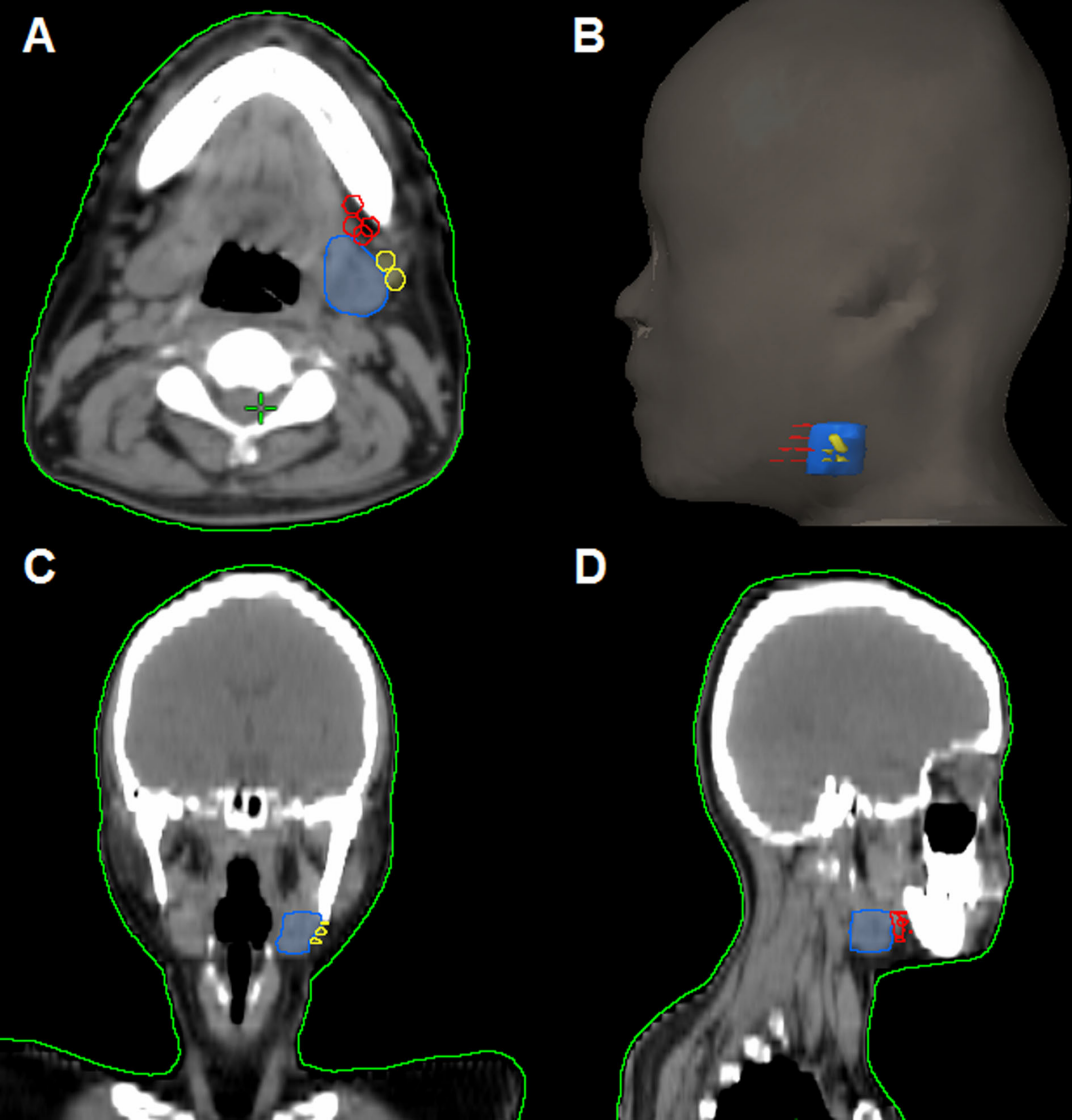
The patterns of peri-submandibular lymph node metastases in axial **(A)**, three-dimensional **(B)**, coronal **(C)**, and sagittal views **(D)** (blue, submandibular gland; red, lymph node metastases in the anterior part of the submandibular gland; yellow, lymph node metastases in the lateral part of the submandibular gland).

**Figure 4:**
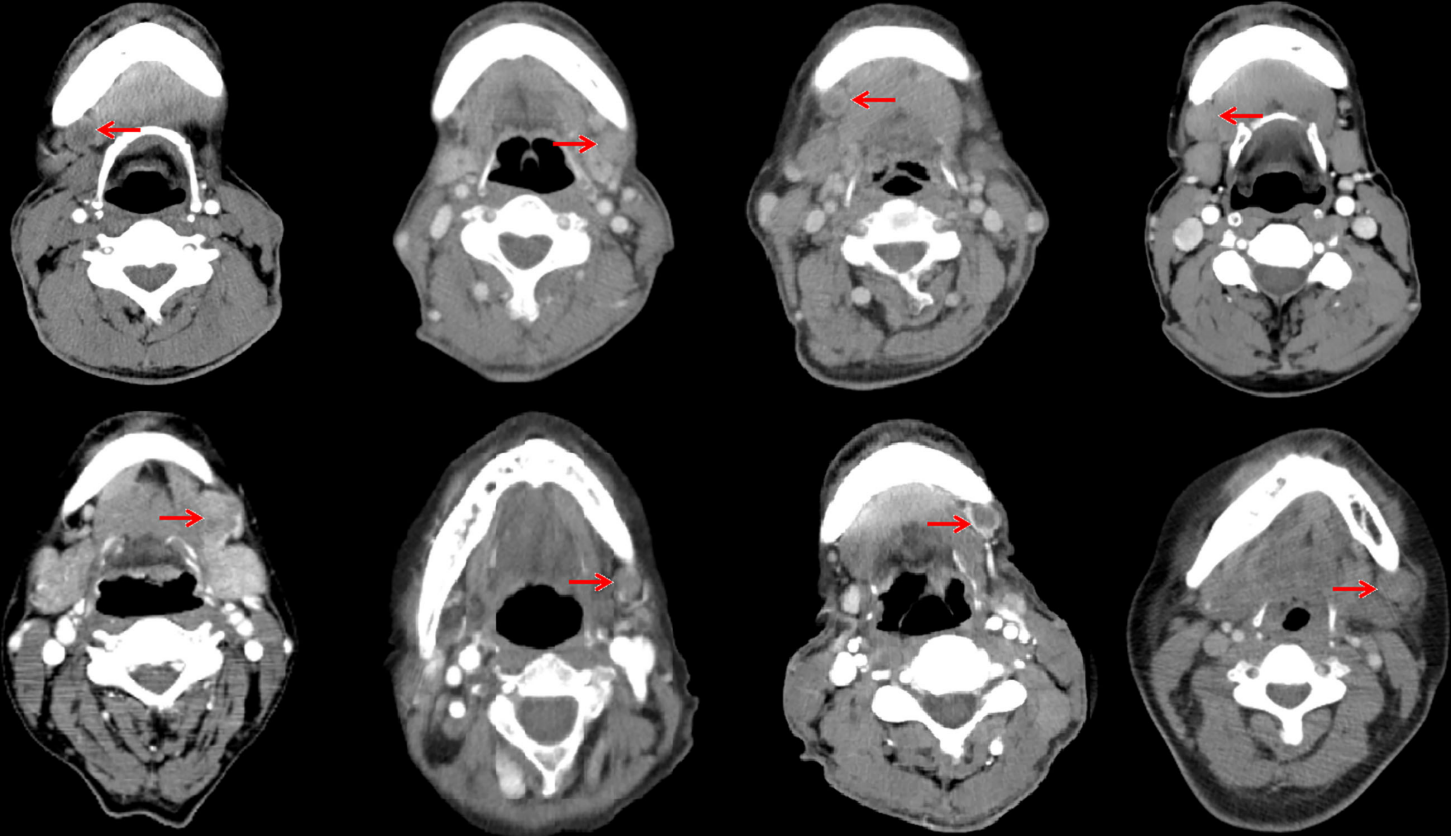
Computed tomography axial images from patients with lymphadenopathy in level IB (red arrow, lymphadenopathy in level IB).

In the 61 patients with pathologically nodal positive diseases, level II was the most common site of regional lymph node metastasis (*n* = 57), followed by level III (*n* = 21), level IB (*n* = 16), level IA (*n* = 2), and level IV (*n* = 1). A total of 18 positive lymph nodes were found in level IB in 16 patients. The median maximum diameter of the positive lymph nodes around level IB was 1.49 cm (range, 1.17–2.61 cm). The patterns of peri-submandibular lymph node metastases are displayed in **Figures 2**–**4**. There were 6 lymph nodes in the lateral part of the SMG, and 12 were shown in the anterior of the SMG. However, no positive lymph node was observed in the medial or internal side of the SMG.

### Risk Factors Associated With Level IB Lymph Node Metastasis

The logistic regression test was performed to determine the predictive factors associated with level IB lymph node metastasis (**Table 2**). The results showed that patients with tumors located in the buccal mucosa (the odds ratio [OR] for buccal mucosal cancer compared to tongue cancer was 6.852, 95% confidence interval [CI] 1.375–34.144, *p* = 0.019) and N3 stage (the OR for stage N3 disease compared to stage N1 disease was 13.333, 95%CI 1.321–134.615, *p* = 0.028) were the independent predictive factors associated with level IB lymph node metastasis.

**Table 2:** Independent predictive factors associated with level IB lymph node metastasis in patients with node-positive disease (*n* = 61).

Variables	OR	95% CI	*p*
Gender			
Male	1		
Female	0.418	0.104–1.689	0.221
Age			
<50 years	1		
≥50 years	0.553	0.138–2.217	0.403
Primary sites			
Tongue	1		
Buccal mucosa	6.852	1.375–34.144	0.019
Others	1.644	0.273–9.892	0.587
Alcohol use			
No	1		
Normal	1.971	0.527–7.374	0.313
Abuse	0.821	0.179–3.374	0.800
Smoking pack-year index			
0	1		
<20	1.667	0.329–8.434	0.537
≥20	0.741	0.194–2.830	0.661
Tumor differentiation			
Well differentiation	1		
Moderate differentiation	0.455	0.066–3.113	0.422
Poor differentiation	0.667	0.078–5.678	0.711
Tumor stage			
T1	1		
T2	0.913	0.193–4.330	0.909
T3	0.667	0.089–4.994	0.693
T4	3.000	0.447–20.153	0.258
Nodal stage			
N1	1		
N2	2.414	0.263–22.117	0.436
N3	13.333	1.321–134.615	0.028
Margin status			
Negative	1		
Positive	0.933	0.090–9.677	0.954

OR, odds ratio; CI, confidence interval; T, tumor; N, nodal.

## Discussion

In our study, we aimed to investigate the rationality of SMG-sparing neck dissection among patients with OSCC. Our study showed that only one patient (0.7%) had SMG involvement, and direct involvement was the most common way of SMG involvement in OSCC patients.

Although the neck dissection procedure has undergone several improvements, the SMG dissection was always recommended in OSCC (14, 15). In recent years, a growing number of evidence showed that the preservation of non-invaded SMG may be feasible in OSCC (8, 9, 16). There are three potential patterns of SMG involvement: anatomic proximity, hematogenous metastasis, and lymphatic spread (17). SMG is thought to lack a blood vessel network, which is different from other glands (17). Although a prior literature review showed a low risk of SMG metastasis in breast, lung, and renal cancers (18), no hematogenous metastasis in SMG was found in OSCC patients (17, 19, 20). In addition, SMG was thought to lack a lymphatic vessel network (17). Zeng et al. made a literature review that included 2,750 patients, and they found that only 0.07% of patients had intraglandular lymph node metastases (8). Furthermore, direct involvement was the main pattern of SMG involvement in OSCC (1%–2.9%) (16, 17, 19, 21). In our study, there was only one (0.7%) OSCC patient who had SMG involvement by direct infiltration from the primary tumor, which was similar to the above studies. Therefore, direct involvement is the most common pattern of SMG involvement in OSCC patients.

SMG is located in level IB according to the current recommendation of neck nodes delineation guideline (7). Fives et al. reported that approximately 44.4% of OSCC patients had pathologically confirmed positive lymph nodes in level I (4). In our study, 61 patients had pathologically nodal positive diseases and 26.2% of them (*n* = 18) had positive lymph nodes in level IB. Although the rate of level IB lymph node metastasis was relatively high in OSCC, the literature review showed that only 2.05% of patients had SMG involvement (8). In our study, no SMG involvement was observed through periglandular nodal extension. Peri-SMG lymph nodes are divided into six subgroups, and the deep groups have fewer lymph nodes, which have little clinical significance (22). In our study, there were 6 lymph nodes in the lateral part of SMG, 12 in the anterior of SMG, and no lymph node was observed in the medial or internal side of SMG. A large cohort of patients with nasopharyngeal carcinoma also showed no patients had SMG metastasis or metastasis to the medial edge of SMG (23). In the current clinical practice, resection of all lymph nodes in level IB and preservation of the SMG are technically feasible for OSCC patients (24). Therefore, with careful preoperative imaging evaluation and intraoperative evaluation of the relationship between primary tumor and metastatic lymph nodes or SMG, SMG-sparing neck dissection may be feasible and safe if the SMG is not involved.

The SMG secretes approximately 70%–90% of the amount of unstimulated salivary, especially at night (25). Saliva plays an important role in oral cavity lubrication, oral antimicrobial activity maintenance, tooth remineralization, and oral mucosal immunity (17). Removal of SMG would increase the incidence of xerostomia and influence the quality of life (17). In addition, in OSCC patients receiving adjuvant radiotherapy, the irradiation of the contralateral SMG could further increase xerostomia because SMG is a part of level IB treatment in the consensus guidelines (7). Moreover, resection of the SMG may also result in external contour defects in the neck (26). Several studies have found that the SMG may be involved by direct invasion of the primary lesion or by spread from adjacent metastatic cervical lymph nodes (21, 27). Advanced T stage and mouth floor tumors were also the risk factors for a direct invasion of SMG (17, 22, 26, 28). In our study, we did not analyze the relationship between clinicopathological factors associated with SMG involvement because limited patients had SMG involvement. We only found one patient with T4N2bM0 oral tongue cancer who had a tumor infiltrated to SMG.

In the recent two decades, there has been controversy over whether SMG needs to be removed in OSCC. With the in-depth understanding of the patterns of lymph node metastases in the neck, selective neck dissection has become widely accepted in the treatment of OSCC. The distribution of lymph node metastases in the neck in our study was similar to the previous studies (29, 30). Since the SMG does not contain intraglandular lymph nodes, removal of an uninvolved SMG may not always be necessary, which has the potential benefit to reduce postoperative xerostomia (26). We also only found one patient with SMG involvement by direct invasion of the primary tumor. The study from Chen et al. showed that stage T4 disease and N2b–N3 tumors were the risk factor for SMG invasion, especially for those with buccal mucosal cancer and cancer located in the alveolar ridge (28). Therefore, the anatomical proximity of primary cancer must be taken into consideration while evaluating the patient for SMG preservation.

According to previous studies, the true infiltration of the SMG by OSCC is quite rare, suggesting that the SMG might not be contaminated and thus be considered to be preserved during level IB lymph node dissection (17, 26, 28). However, we should emphasize the limited insight into the operating field when preserving the SMG during neck dissection, including the risk of nerve injuries and the risk of missing affected lymph nodes (9, 31). In the clinical practice, the protection of SMG may also be safe. Zeng et al. confirmed the oncological safety of SMG flaps in repairing postoperative OSCC defects (8). Moreover, SMG transplantation to the anterior submental region has been found to protect the gland from the dry mouth during radiotherapy (32). Regarding the contemporary radiotherapy techniques, William et al. demonstrated the feasibility of SMG preservation by maintaining a mean dose to the gland of ≤39 Gy (33).

In our study, patients with buccal mucosal cancer and N3 stage have a higher risk of level IB metastasis. However, there was no significant association between T stage and level IB metastasis. Several studies also have shown that the T stage was not a risk factor for level IB metastasis (16, 26, 34).

Several limitations should be acknowledged in the current study. First, it was a retrospective study with relatively small sample size. Second, our study does not include information on actual complaints by patients concerning the removal of SMG. Third, as the follow-up time in our study was relatively short, a long-term observation is required to determine the risk of tumor recurrence in level IB. Finally, the long-term safety of SMG preservation in OSCC should be performed by the prospective studies. Despite these limitations, we believe that our findings add the knowledge regarding the preservation of SMG for OSCC patients.

## Conclusion

In conclusion, our study suggests that the involvement of SMG is extremely rare in OSCC. Preservation of the SMG during neck dissection in selected patients with OSCC seems to be feasible and oncologically safe. More studies are needed to investigate the candidates who may be feasible and safe to preserve SMG.

## Data Availability Statement

The raw data supporting the conclusions of this article is available from the corresponding author on reasonable request.

## Ethics Statement

The study was approved by the Institutional Review Board of the First Affiliated Hospital of Xiamen University. Written informed consent for participation was not required for this study in accordance with the national legislation and the institutional requirements.

## Author Contributions

PZ, J-XC, YZ, BY, and S-GW are lead authors who participated in data collection, manuscript drafting, tables/figures creation, and manuscript revision. PZ and C-LL aided in data collection. PZ, J-XC, and YZ are senior authors who aided in drafting the manuscript and manuscript revision. BY and S-GW are the corresponding authors who initially developed the concept and drafted and revised the manuscript. All authors contributed to the article and approved the submitted version.

## Funding

This work was partly supported by the Key R&D Plan of Hainan Province (No. ZDYF2021SHFZ115).

## Conflict of Interest

The authors declare that the research was conducted in the absence of any commercial or financial relationships that could be construed as a potential conflict of interest.

## Publisher’s Note

All claims expressed in this article are solely those of the authors and do not necessarily represent those of their affiliated organizations, or those of the publisher, the editors and the reviewers. Any product that may be evaluated in this article, or claim that may be made by its manufacturer, is not guaranteed or endorsed by the publisher.
